# Wearable Devices for the Quantitative Assessment of Knee Joint Function After Anterior Cruciate Ligament Injury or Reconstruction: A Scoping Review

**DOI:** 10.3390/s25185837

**Published:** 2025-09-18

**Authors:** Oliwia Ptaszyk, Tarek Boutefnouchet, Gerard Cummins, Jin Min Kim, Ziyun Ding

**Affiliations:** 1 School of Engineering, University of Birmingham, Birmingham B15 2TT, UK; tarek.boutefnouchet@uhb.nhs.uk (T.B.); g.cummins@bham.ac.uk (G.C.); j.m.kim@bham.ac.uk (J.M.K.); z.ding@bham.ac.uk (Z.D.); 2 Institute of Clinical Sciences, University of Birmingham, Birmingham B15 2TT, UK; 3 University Hospitals of Birmingham, Birmingham B15 2GW, UK

**Keywords:** anterior cruciate ligament (ACL), wearable devices, knee biomechanics, inertial measurement units (IMUs), clinical translation, sensor validation, technology readiness level (TRL)

## Abstract

Anterior cruciate ligament (ACL) injury and reconstruction (ACLR) are associated with biomechanical deficits and reinjury risk. Wearable devices offer promising tools for objective assessment of knee joint function. This scoping review aimed to map the use of wearable devices in quantifying knee outcomes following ACL injury or reconstruction, and to evaluate their clinical readiness and methodological quality. Eligible studies were human, English-language studies in ACL/ACLR populations or healthy cohorts assessing ACL-relevant knee outcomes with wearable devices. MEDLINE (Ovid), Embase (Ovid), APA PsycInfo (Ovid), PubMed, and Scopus were searched up to 27 August 2025. Data on devices, tasks, participants, outcomes, and validation were extracted, and an adapted technology readiness level (TRL) mapping was applied. Thirty-two studies met the inclusion criteria. Inertial measurement units (IMUs) were used most often for kinematics. Standalone accelerometers quantified pivot-shift features, while force-sensing insoles captured bilateral loading. Electromagnetic trackers and electrogoniometers served as higher-precision comparators but were workflow-limited. Reporting of calibration and criterion validation was inconsistent. TRL bands clustered at 3–6, and none reached clinical integration. We propose task-matched sampling, transparent calibration, criterion validation, pairing with patient-reported outcome measures (PROMs), and multi-site workflow trials to progress towards routine care.

## 1. Introduction

**Figure 1:**
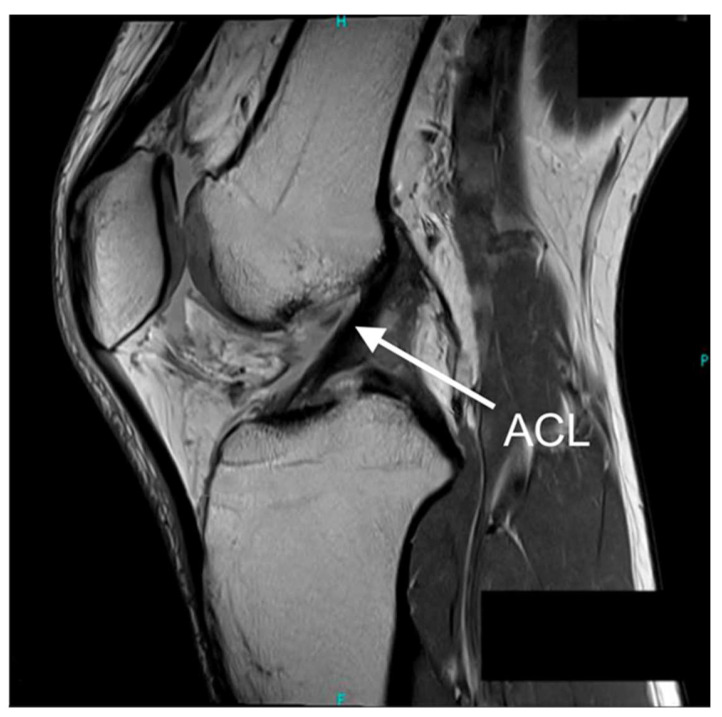
Sagittal view MRI (magnetic resonance imaging) scan of the knee joint. Intact ACL indicated by the arrow, demonstrating normal ligament continuity and positioning. Orientation labels: H—head (superior), F—foot (inferior), P—posterior. Adapted from [3], CC BY 4.0. Changes: original box annotation and panel label removed.

The anterior cruciate ligament (ACL) is essential for maintaining knee stability, primarily by restricting anterior tibial translation and secondarily by rotational control [1]. Anatomically, the ACL consists of two distinct bundles—the anteromedial (AM) and posterolateral (PL), which contribute to knee stability throughout its entire range of motion (ROM). These bundles originate from the posteromedial aspect of the lateral femoral condyle and attach to a wide, oval-shaped area on the tibia, located centrally and slightly anterior to the intercondylar eminences (Figure 1) [1]. This anatomical location enables the ACL to accommodate complex loading patterns. Biomechanically, the ACL demonstrates significant tensile strength, with an average ultimate load to failure of approximately 1725 ± 269 N [2]. The ACL’s structure, positioning, and mechanical strength highlight its critical function in stabilising the knee during dynamic movements such as pivoting, cutting, and deceleration.

Following the ACL rupture, this structural integrity is compromised, leading to increased joint laxity and instability. This is characterised by excessive anterior tibial translation and abnormal rotational movement [4], which are commonly assessed using manual clinical tests such as the Lachman test (primarily evaluating anteroposterior laxity), and the pivot-shift test (used to determine anterolateral rotational laxity) [5]. According to a meta-analysis by Benjaminse et al. [5], when performed without anaesthesia, the Lachman test demonstrated the highest diagnostic accuracy for detecting ACL ruptures, with a pooled sensitivity of 85% and specificity of 94%. The pivot-shift test, while highly specific (98%), had a notably low sensitivity (24%) under the same conditions, limiting its utility in ruling out ACL injuries. The anterior drawer test also assesses anteroposterior laxity, but shows lower diagnostic consistency (sensitivity of 55%, specificity of 92%). Its utility in ACL rupture diagnosis in acute injuries was recognised as questionable due to joint effusion, pain, and hamstring guarding. Manual clinical examination tests are operator dependent and vary in their susceptibility to muscle guarding, especially in the acute setting [5]. Hop tests have been developed as field-based assessments to evaluate knee functionality following ACL reconstruction (ACLR). By comparing the height and distance of hops, these tests identify asymmetries between operated and non-operated limbs. However, they provide limited insight into the underlying movement mechanics or pathological laxity [6]. Therefore, there is a need for the further development of objective assessment methods to evaluate knee joint function after ACL injury or reconstruction.

In addition to its mechanical role, the ACL contributes to proprioception through embedded mechanoreceptors, such as Ruffini endings and Golgi tendon organs, which provide sensory feedback on joint position. Barrack et al. demonstrated significantly impaired proprioceptive function in patients with complete ACL tears, highlighting its role in neuromuscular control [4]. Georgoulis et al. further showed that proprioceptive deficits following ACL rupture can alter gait patterns and contribute to long-term joint degeneration, highlighting both the mechanical and sensory function of the ACL [7].

The ACL is one of the main stabilising structures in the knee. Its rupture can lead to instability, pain, loss of function, and an increased risk of secondary injuries, especially to the menisci [8]. The ACL is the most commonly injured ligament in the knee, with an estimated 400,000 ACLRs performed each year worldwide, making it the third most frequent elective orthopaedic intervention in developed countries [9,10,11,12]. In England, it is estimated that approximately 15,000 primary ACLRs are performed annually [13]. This figure is likely an underestimation, being based on Hospital Episode Statistics (HES) data. The incidence of ACL surgery has increased significantly over the last 20 years [9,14], with a rising incidence in both females and those younger than 20 years old [15]. Females are at significantly higher risk of ACL injury than males, with incidence rates reported to be up to eight times greater in comparable sports activities [16]. ACL surgery remains the preferred treatment choice in active patients who wish to return to jumping, cutting, or pivoting activities. It involves reconstructing the torn ligament using a graft, which passes through anatomically positioned bone tunnels in the femur and tibia to restore knee stability. Various graft types are used, including hamstring tendon, bone–patellar tendon–bone (BPTB), quadriceps tendon, and allografts from donor tissue [17]. The timing of ACLR has been a source of debate [17,18], but available evidence supports a judicious period of delay before surgical intervention until the knee is considered ”quiet” following the resolution of the initial inflammatory phase [19]. ACL reconstruction has also been shown to be cost-effective, expressed as quality-adjusted life years (QALYs) gained, and driven by the higher probability of an unstable knee in the rehabilitation alone management [20,21].

Moreover, ACLR does not guarantee a return to pre-injury function. Only 65% of patients who undergo ACLR return to pre-injury levels of sport participation [22]. Significant biomechanical discrepancies between limbs have been identified following ACLR, which are strongly correlated with an increased risk of reinjury [23]. The risk of sustaining a secondary knee injury, in the contralateral knee or the reconstructed ACL, has been estimated at 29% following primary ACLR [24]. Thus, it is crucial to advance the methods used to assess clinical and functional outcomes following ACLR.

**Figure 2:**
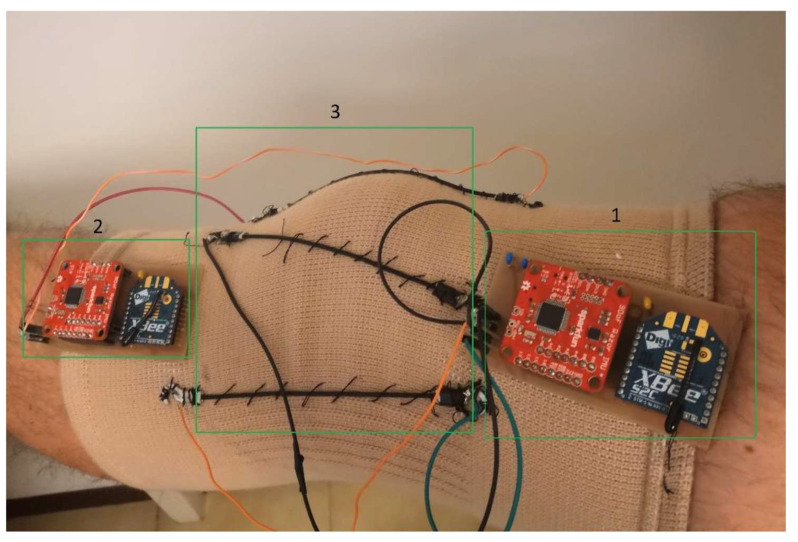
Smart knee brace for dynamic laxity measurement. (1) Femoral unit integrating an IMU, microcontroller and wireless transmitter; (2) tibial unit with IMU, microcontroller and transmitter; (3) stretch sensors sewn into the sleeve to capture translational and rotational laxity during Lachman/anterior drawer/pivot-shift tests. Adapted from [26], CC BY 4.0, original annotations retained.

Wearable devices are technologies worn on the body or integrated into clothing that include sensors to detect physiological, biochemical, mechanical, or environmental signals (Figure 2). These devices may transmit data to external systems for processing or include embedded electronics for local analysis [25]. Wearable devices, including inertial measurement units (IMUs), accelerometers, force-sensing insoles, and strain sensors, offer portable and increasingly cost-effective solutions for measuring biomechanics outside of laboratory environments. Their potential to assess knee joint kinematics and kinetics in both clinical and field settings makes them highly relevant for monitoring recovery, identifying risk factors for re-injury, and guiding rehabilitation.

Although several studies have explored wearable devices in this context, many lacked detailed reporting of device specifications, study protocols, and validation metrics, and few applied formal frameworks to technology readiness for implementation. These challenges limit the ability to draw meaningful conclusions, delay their transition into routine clinical practice, and leave healthcare professionals uncertain about their real-world application in patient care.

This review provides a comprehensive and updated synthesis of how wearable devices can be used to objectively assess knee joint function and monitor outcomes following ACL injury or reconstruction. By addressing these key aspects, this review aims to enhance the clinical utility of wearable devices, improve diagnostic accuracy, and optimise rehabilitation strategies. Ultimately, this could lead to better patient outcomes, more personalised treatment approaches, and reduced healthcare costs associated with knee injuries and rehabilitation.

Recent reviews describe heterogeneous sensor setups and custom analysis pipelines that hinder cross-study comparison. Most ACL wearable studies remain proof-of-concept with limited clinical adoption [27,28]. Multiple reviews call for rigorous criterion validation, clearer reporting (e.g., calibration and reliability methods), and multi-centre trials, noting that current metrics are not tied to predictive or decision-support value in routine care [27,28,29]. This scoping review addresses those gaps by synthesising 32 studies across IMUs, accelerometers, electromagnetic tracking, insoles, and electrogoniometers, and by distinguishing directly measured outcomes from model-estimated surrogates. It applies an adapted technology readiness level (TRL) mapping and evaluates validity reporting (in-study criterion, prior-only, or not reported) to describe translational maturity. It also summarises task-specific sampling ranges and protocol features reported in the literature (e.g., higher rates for impact kinetics and moderate rates for gait/stairs) linked to the signals of interest. In addition, the review highlights core reporting elements (calibration routine, trial handling, participants, criterion reference with accuracy and reliability metrics) that would support a minimum reporting set for future ACL wearable studies. Finally, it categorises commonly reported metrics with potential clinical relevance, such as pivot-shift, posterior tibial acceleration and Lachman translation for laxity grading, and insole-derived peak impact force and loading rates as limb-symmetry indices during rehabilitation. To the authors’ knowledge, this is the first ACL-focused review to combine TRL mapping, study-level validation reporting, and cross-device comparison of protocol patterns.

## 2. Materials and Methods

This review followed the Arksey and O’Malley framework [30] and the PRISMA-ScR (Preferred Reporting Items for Systematic Reviews and Meta-Analyses extension for Scoping Reviews) guidelines [31]. The protocol was registered with the Open Science Framework (OSF) Registries (https://osf.io/sngbz (accessed on 9 September 2025)). First, a preliminary scoping search of PubMed and Scopus was conducted in April 2025 to map the field and capture early candidate records. MEDLINE (Ovid), Embase (Ovid), APA PsycInfo (Ovid), PubMed, and Scopus were searched up to 27 August 2025. We used both the acronym “ACL” and the full term “anterior cruciate ligament”, alongside measurement and wearable device terms, with no date, language, or study-design filters. The core free-text query was as follows: (ACL or “anterior cruciate ligament”) and (injur* or reconstruct*) and (assess* or model* or measure* or estimat* or quantif*) and (wearable or sensor or sensors) and outcome. No additional filters were applied. The search strategy was developed by two authors (Z.D., T.B.), who have expertise in scoping review methodology and orthopaedic research.

Eligibility criteria were defined using the population, concept, and context (PCC) framework [32]. The population included individuals of any age, gender, ethnicity, or activity level with a history of ACL injury or reconstruction, as well as healthy participants, where ACL-related outcomes were assessed. The concept required a wearable device to quantify a knee joint outcome. We also included wearable-derived outcomes of knee loading (e.g., insole plantar-load metrics) when used to assess ACL-relevant tasks and supported by prior validation or rationale linking them to knee loading or symmetry after ACL injury. Studies reporting only temporospatial or performance metrics (e.g., gait cycle time, stance/swing time, cadence, jump height) were excluded unless such metrics were explicitly justified or previously validated as proxies of knee loading or symmetry in ACL-relevant contexts. Studies reporting only physiological signals (e.g., electromyography/electroencephalography/near-infrared spectroscopy) without a wearable-derived knee variable were excluded. The context was limited to human studies reported in English. Review articles, commentaries, qualitative studies, and cadaveric or animal studies were excluded.

Titles, abstracts and full texts were screened independently by three reviewers (O.P., Z.D., J.K.), with disagreements resolved by discussion. No authors were contacted for clarification. Data was entered in a predefined Excel form (study design; cohort characteristics; device/placement; sampling and calibration/protocols; outcomes; accuracy statistics). Each study was tagged with a validation reference code indicating whether any wearable-derived knee outcome in that paper underwent an in-study criterion comparison (I), relied on prior-literature validity without in-study criterion (P), or did not report criterion validity (N). When multiple outcomes were present, the code reflects the strongest level achieved in that study. Results were synthesised narratively, with visual summaries of device use, participant demographics, and key outcomes. We classified technology maturity using a descriptive TRL mapping applied at the device/system level, rather than relying on single-number TRLs. This mapping groups UK Research and Innovation (UKRI) TRL bands into four qualitative categories summarised in Table 1 [33]. For each device, we recorded concise evidence justification. Since eligibility required a functioning wearable system that generated quantitative knee outcomes, studies at TRL 1–2 (basic discovery or concept only, without a working device) were not eligible. Consequently, our maturity mapping begins at TRL 3. Validation metrics and adoption indicators were extracted and reported separately, and did not contribute to the maturity category.

## 3. Results

**Figure 3:**
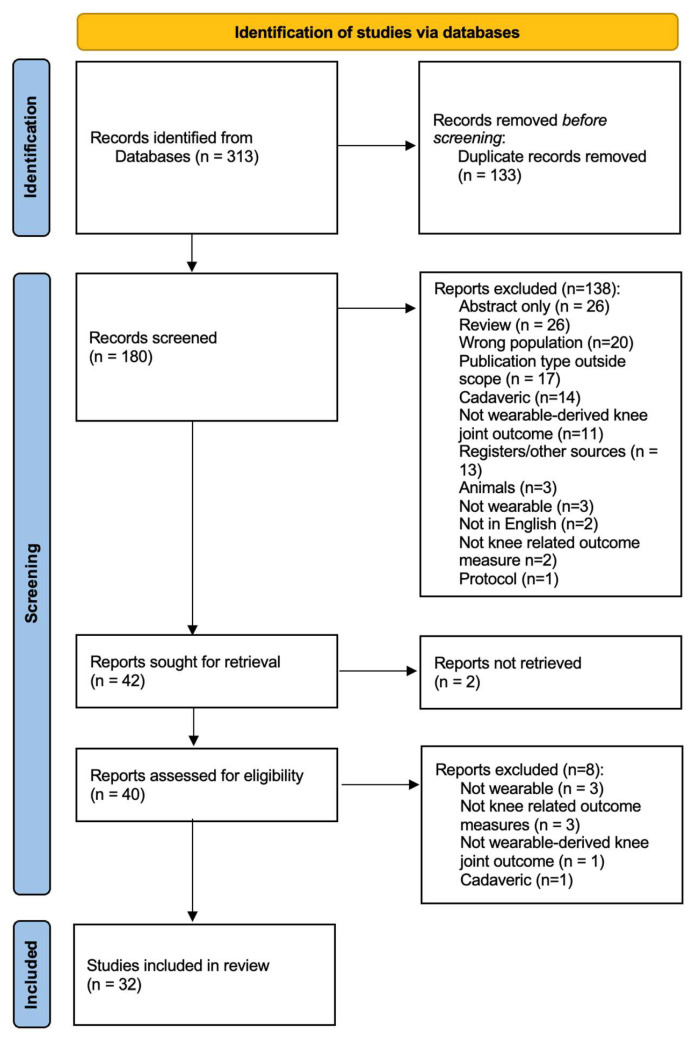
PRISMA-2020 flow diagram of study selection.

The electronic searches identified 313 records. After de-duplication (133 removed), 180 records were screened by title/abstract. We excluded 138 records at this stage: abstract only (*n* = 26), review (*n* = 26), wrong population (*n* = 20), publication type outside scope (*n* = 17), cadaveric (*n* = 14), not wearable-derived knee joint outcome (*n* = 11), registers/other sources (*n* = 13), animals (*n* = 3), not wearable (*n* = 3), not in English (*n* = 2), not knee-related outcome measure (*n* = 2), and protocol (*n* = 1). We sought 42 reports for retrieval; 2 were not retrieved. We assessed 40 full texts for eligibility and excluded 8 (not wearable, *n* = 3; not knee-related outcome measures, *n* = 3; not wearable-derived knee joint outcome, *n* = 1; cadaveric, *n* = 1). In total, 32 studies were included in the review (Figure 3). Descriptive characteristics of the included studies are provided in the main text and Supplementary Material File S1.

### 3.1. Device Details

A range of wearable devices was used across the included studies, varying in type, configuration, placement, and sampling frequency. Devices were grouped into the following categories: inertial measurement units (IMUs), accelerometers, electromagnetic sensors, force-sensing insoles, and others (electrogoniometer, inductive displacement sensor). Table 2 summarises key specifications, with full details in the Supplementary Material File S1.

#### 3.1.1. Inertial Measurement Units (IMUs)

IMUs are compact, non-invasive devices that typically combine accelerometers, gyroscopes, and sometimes magnetometers to measure linear acceleration, angular velocity, and orientation. They are widely used in biomechanics due to their portability, affordability, and suitability for both laboratory and real-world environments. IMUs are often embedded in wireless systems with onboard telemetry, allowing untethered data collection via Bluetooth or other wireless protocols. However, their accuracy can be affected by soft tissue artefact [66] and sensor drift over time [67]. IMUs are generally accurate for tracking human joint angles and movement patterns, especially in simple, single-plane tasks. In their systematic review, Poitras et al. reported that IMUs estimate knee joint angles with root mean square errors (RMSE) ranging from 1° to 11.5° and intraclass correlation coefficients (ICCs) between 0.4 and 1.00, with most values exceeding 0.80 for flexion and extension movements [68].

IMUs were the most commonly used device, appearing in half of the reviewed studies. Sensor placement varied depending on the study objectives, with thigh and shank being the most used locations [26,36,37,39,40,41,45,48]. Additional placements included the feet, pelvis/sacrum, trunk/chest, and upper limbs for full-body or alternative motion analysis. One study combined IMUs with stretchable strain sensors in a smart brace [26].

The number of IMUs used ranged from 1 to 17, with simpler configurations involving up to 4 sensors [26,38,39,40,41,42,43,44,46,47,48], and more complex setups using 7 or more [34,35,36,37,45]. Sampling rates also varied, with many studies using 40–100 Hz [26,35,39,40,45], and others employing higher frequencies such as 150 Hz [41], 240 Hz [36,48], and 500 Hz [38,46]. Notably, Niederer et al. [43] reported exceptionally high sampling rates ranging from 4.5 to 9.0 kHz. Several studies did not report sampling frequency [34,37,42,44,47].

Signal processing techniques varied across studies, including time-series filtering, gait event detection, and machine learning classification, often implemented using custom-written MATLAB or Python scripts [26,36,37,38,40]. In contrast, others employed dedicated software such as MVN BIOMECH Studio [37] or the Orthelligent HOME app [44]. Dowling et al. [48] implemented a custom feedback system based on inverse dynamics and point cluster techniques, while Ahmadian et al. [46] used custom software tailored to different Physilog models.

#### 3.1.2. Accelerometers

Accelerometers are sensors that measure linear acceleration along one or more axes. While they are often integrated into inertial measurement units (IMUs), standalone accelerometers can quantify tibial acceleration during high-velocity tasks (e.g., the pivot-shift), or be combined with image-based analysis to estimate translation. These devices are lightweight, low-cost, and easy to implement in intraoperative or clinical settings, but they lack the rotational data provided by gyroscopes and magnetometers, which limits their ability to capture full joint kinematics.

Triaxial accelerometers were used in three studies [18,49,51]. Lopomo et al. [51] used an accelerometer mounted on the tibia to quantify tibial acceleration during the pivot-shift test. Diermeier et al. [49] incorporated an accelerometer into a hybrid system combining inertial sensing with image-based analysis to evaluate lateral compartment translation, and Musahl et al. [50] used it to quantify tibial acceleration and paired it with tablet-based video analysis for translation.

#### 3.1.3. Electromagnetic Sensors

Electromagnetic sensors track the position and orientation of body segments using magnetic fields. Electromagnetic tracking systems demonstrated sub-millimetre to low-millimetre spatial accuracy in controlled laboratory settings, with standardised reporting assessments reporting position errors below 1.2 mm for distances below a few centimetres, corresponding to relative errors of up to 2.4% [69]. However, they are sensitive to magnetic interference and typically require wired setups, which can limit their use in dynamic or cluttered clinical environments [69]. While wireless sensor options do exist, they tend to be bulkier and more complex due to the need for onboard power and active transmission, which can introduce additional constraints [69].

This technology was used in five studies [52,53,54,55,57], primarily to assess tibiofemoral kinematics and quantify pivot shift. Sensor placement typically involved the tibia and femur, with sensors positioned approximately 7–10 cm below the tibial tubercle and 10–13 cm above the patella [54,55]. Most studies used two to three sensors, while Kuroda et al. [54] used an additional sensor for digitising anatomical landmarks. Sampling rates ranged from 40 Hz [57] to 100 Hz [52], with 60 Hz being the most common [54,55]. Data were typically processed using 6 degrees of freedom (DOF) kinematic models, such as the joint coordinate system by Grood and Suntay [54,55]. Specific software platforms were not always disclosed.

#### 3.1.4. Force Sensing Insoles

Force-sensing insoles are embedded in footwear and use capacitive sensors to measure vertical ground reaction forces during movement. They are particularly useful for assessing gait patterns, landing mechanics, and limb loading asymmetries. As non-invasive and user-friendly systems, they offer a practical alternative to laboratory-based equipment. Notably, they have demonstrated good to excellent reliability, with intraclass correlation coefficients (ICCs) ranging from 0.88 to 0.96 when validated against gold-standard measurement systems [70]. In terms of accuracy, Seiberl et al. reported a mean bias of less than 4% for key parameters such as ground contact time, impulse, peak force, and time to peak, with 95% of measurement differences falling within 12% of force plate values [71]. Compared with force plates, insoles show a small underestimation bias [62]. Footwear standardisation can also influence results, and insoles typically record only the vertical component [60,72].

Five studies employed this technology using the Loadsol system [58,59,60,61,62]. In all cases, the insoles were placed under each foot, with one sensor per foot, allowing for bilateral assessment of loading patterns. Sampling rates were typically 100 Hz [58,59,60,61,62], although Peebles et al. [62] and Luftglass et al. [60] also used a higher rate of 200 Hz for more detailed analysis during landing tasks. Data were processed using the MATLAB [60,62], Loadsol mobile application [58,59] and custom MATLAB-based programs developed by Peebles et al. [58,59].

#### 3.1.5. Other Devices

Two studies used a single electrogoniometer to measure joint-position sense (JPS) in ACLR groups. Their protocols were closely related, and both studies collected data at 4 kHz, and used IMAGO Process Master for acquisition and Excel for post-processing [63,64]. Electrogoniometers offer a simple setup with high sampling rates; however, they are limited by a single-plane measurement and soft-tissue artefacts [73].

Deiss et al. [65] implemented an inductive displacement sensor to quantify anterior tibial translation under both loaded and unloaded conditions. Sensors were positioned on the patella and tibial tuberosity, offering a direct mechanical measure of joint laxity. This approach provided a high-resolution alternative to traditional manual assessments, with a measurement sensitivity of 0.60–0.69 mm and a minimum detectable change (MDC) between 1.67 and 1.96 mm during passive Lachman testing. These values indicate the system’s ability to reliably detect anterior tibial translation differences within clinically relevant thresholds, such as the 3–5 mm range often used to identify ACL deficiency [74,75].

Together, these studies highlight the expanding diversity of wearable devices in ACL research, and highlight the potential for integrating novel sensors into broader biomechanical and clinical assessments.

### 3.2. Participants’ Demographics

**Figure 4:**
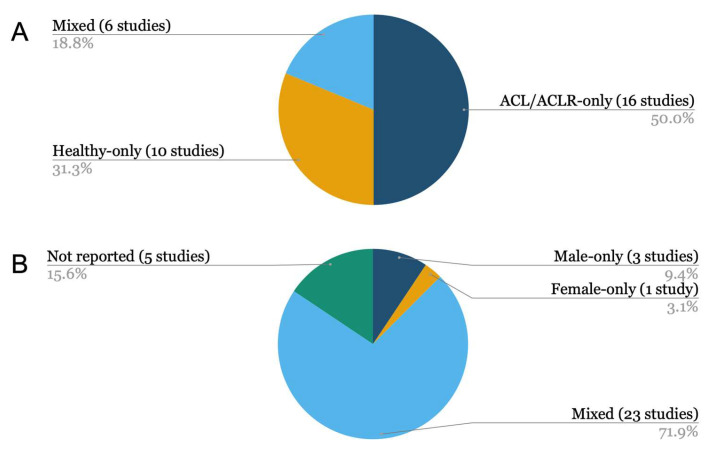
Participants at a glance (*n* = 32 studies). (**A**) Cohort type: ACL/ACLR-only, 16 (50%); Mixed ACL-controls, 6 (18.8%); Healthy-only, 10 (31.3%). (**B**) Sex makeup by study: Mixed-sex, 23 (71.9%); Male-only, 3 (9.4%); Female-only, 1 (3.1%); Sex not reported, 5 (15.6%). Note: Percentages may not sum to 100% due to rounding.

The studies included in this scoping review involved a diverse range of participants, both in terms of demographics and clinical status. Sample sizes varied considerably, ranging from small exploratory cohorts of four participants [26,40] to large registry-based datasets involving over 1400 individuals [43]. Because participant sex can modify ACL injury risk and key biomechanical outcomes captured by wearables (e.g., landing mechanics or rotatory laxity features), we summarise sex distribution alongside cohort composition to contextualise the generalisability of sensor-derived findings. Figure 4 provides a compact visual summary.

#### 3.2.1. Participants’ Groups

To contextualise the generalisability of wearable-derived outcomes, we classified cohorts into three groups:

- ACL/ACLR-only,
- Mixed, that is ACLR/ACL-deficient (ACLD) with healthy controls,
- Healthy-only.

Across the 32 included studies, ACL/ACLR-only cohorts were most common (16/32; 50%) [26,37,41,42,43,44,47,49,50,51,53,54,55,57,58,59], followed by Healthy-only (10/32; 31.3%) [35,36,38,39,45,48,52,60,62,65] and Mixed cohorts (6/32; 18.8%) [34,40,46,61,63,64] (see Figure 4A). In ACL/ACLR-only studies, wearables were typically used to quantify rotatory laxity (e.g., quantitative pivot-shift) or post-operative function (e.g., gait/landing symmetry). Mixed designs included healthy controls as a reference to interpret deficits or recovery. At the same time, Healthy-only studies predominantly addressed device validation, risk-factor screening, or model development (e.g., estimating kinetics from IMUs).

Since several outcomes assessed in this review (landing mechanics, joint-loading surrogates, and rotatory-laxity features) are sex-sensitive, we present sex distribution alongside this cohort grouping in Section 3.2.2 and Figure 4B to aid interpretation and external validity.

#### 3.2.2. Demographics

Across the included studies, participant demographics varied in terms of age, sex, height, weight, and body mass index (BMI), reflecting the variety of populations investigated for ACL injury or reconstruction outcomes. Reported ages ranged from adolescents as young as 13 years [58,59] to adults over 40 years [43,49,51], with mean or median ages most frequently falling between 20 and 30 years. For example, the cohort in Mengis et al. [44] reported a median age of 25.3 years, while Peebles et al. [62] included participants with a mean age of 22.2  ±  3.4 years. In contrast, Labbé et al. [41] and Benjaminse et al. [36] included teenage participants with a mean age of 15.5  ±  1.5 years and 14.8  ±  1.0 years, respectively.

Sex distribution varied; many used mixed-sex samples, while three included only male participants [38,39,45], only one exclusively female cohort [36], and five did not report sex [26,37,42,49,53] (Figure 4). We highlight sex because females have higher ACL injury risk [16], and several wearable outcomes in this review are sex-sensitive constructs (e.g., landing and hop kinetics from insoles, IMU-estimated vertical ground reaction force (vGRF) or knee extension moment (KEM) during landings, and knee abduction moment classification during cutting) [76,77,78]. Thus, sex balance directly affects external validity and comparison across studies.

Height and weight/BMI were reported in 18 studies. Most participants fell within the normal to overweight BMI range. For example, individuals in the study by Kawanishi et al. [47] had an average BMI of 22.6, while Mengis et al. [44] reported a median BMI of 23.9. The variability in demographic reporting highlights the importance of standardising participant descriptions in future wearable device studies

#### 3.2.3. Activity Level and Athletic Background

Activity levels were often defined using standardised scales such as the Tegner Activity Score [35,38,44,63,64,65] or the Marx Activity Rating Scale [61]. Participants ranged from non-active individuals to professional athletes [35,36]. Several studies specifically targeted recreational athletes involved in jumping or cutting sports [38,48].

#### 3.2.4. Exclusion Criteria

Most studies employed specific exclusion criteria to ensure consistency within their study populations. Commonly applied exclusion criteria included the following:

- Previous ACL reconstruction or other major ligamentous surgery on the affected limb [34,44,49,50,51,55,58,59];
- Concomitant injuries such as meniscal tears, collateral ligament injuries, or significant cartilage damage [34,44,47,49,58,59];
- History of lower extremity fractures, neurological impairments, or other musculoskeletal disorders [34,39,42,44,45,46,58,59,63,64,65];
- Current pregnancy [43,44,62,63,64];
- For healthy controls: any history of knee injury, surgery, or instability [35,38,39,46,48,60,61,62].

While exclusion criteria were generally consistent across studies, not all authors reported them in detail.

### 3.3. Task Protocols

The task protocols varied widely across the studies in terms of setting, calibration procedures, task design, and repetition schemes, reflecting the diverse research aims and sensor systems used. These protocols can be broadly categorised into three domains: joint laxity assessments, dynamic functional tasks, and rehabilitation or controlled loading protocols.

#### 3.3.1. Joint Laxity Assessments

Knee laxity assessments, such as the Lachman, anterior drawer, and pivot-shift tests, are commonly used to evaluate the mechanical stability of the knee joint. These tests assess different aspects of tibiofemoral translation and rotation, with the Lachman test being highly sensitive for anterior tibial translation, and the pivot-shift test being highly specific for anterolateral rotatory instability. The anterior drawer test, while less sensitive in acute injuries, remains a valuable tool for detecting anterior laxity in chronic ACL-deficient knees [5].

In the reviewed studies, pivot-shift manoeuvres were predominantly conducted in clinical or intraoperative settings to quantify anterolateral rotatory laxity, often under general anaesthesia or immediately before and after ACL reconstruction. These protocols typically involved repeated pivot-shift manoeuvres using IMUs [26,41,42,47], accelerometers [49,50,51], or electromagnetic sensors [53,54,55,57]. In addition to the pivot-shift test, Bellitti et al. [26] also performed Lachman and anterior drawer tests to assess knee laxity quantitatively. Similarly, Deiss et al. [65] employed Lachman testing and external perturbation-based loading to evaluate anterior tibial translation using the inductive displacement sensor. The number of repetitions ranged from one to nine per limb, with some studies selecting the highest tibial acceleration [55] or analysing only the middle three [47]. Calibration procedures were generally not reported in these studies, likely due to the constraints of the surgical environment.

#### 3.3.2. Dynamic Functional Tasks

In contrast, dynamic functional tasks were primarily conducted in laboratory settings and included activities such as drop jumps, hops, cutting manoeuvres, and stair assessments. These tasks were designed to evaluate functional performance, limb asymmetry, and neuromuscular control. They simulate real-world movements and are used to identify deficits in coordination, balance, joint stability and compensatory movement patterns that may not be detected during static assessments. Drop landing protocols typically involved bilateral or unilateral jumps from 30 to 36 cm platforms, with three to seven successful trials per condition [39,43,45,48,59,60]. Dowling et al. [48] collected data across three sessions and incorporated a motor learning approach with feedback, which resulted in up to 26 trials per participant. Hop tests, including single, triple, and crossover hops, were usually preceded by warm-up or practice sessions and repeated two to seven times per leg to assess knee joint kinematics, symmetry and temporospatial parameters [38,43,46,61,62]. Peebles et al. [62] also included a stop jump task, in which participants sprinted forward and performed a running vertical jump, providing additional insights into dynamic landing strategies, and Niederer et al. included a joint position sense task to evaluate sensorimotor function. Cutting tasks [35,36,39] involved unanticipated or preplanned sidestep movements with randomised direction and rest intervals. Stair negotiation was analysed in two studies: ascent and descent over a 20-step flight using seven IMUs at 3 and 5 months post-ACLR [34], and stair ascent acquired within a clinical IMU toolkit [37]. Gait assessments were performed during overground or treadmill walking at self-selected speeds, using IMUs [37,40] and force-sensing insoles [58]. Only one study [58] explicitly reported a minimum of 40 gait cycles per trial. Others varied in reporting: Button et al. [37] conducted overground gait and stair ascent analysis without specifying trial duration or stride count, and Albano et al. [40] recorded four 2-min treadmill trials.

#### 3.3.3. Rehabilitation, Joint Position Sense (JPS) and Loading Protocols

Rehabilitation protocols are structured interventions designed to restore strength, mobility, and neuromuscular control after injury [79]. In contrast, loading protocols refer to a structured application of mechanical load, such as tension, compression, or shear, on musculoskeletal tissues to stimulate adaptation or assess biomechanical responses [80]. Mengis et al. [44] used a sensor-based mobile application to monitor rehabilitation progress through periodic tests of joint range of motion, coordination, and functional performance at designated follow-up points during outpatient rehabilitation. Two studies utilised joint position sense tasks to assess proprioception using active angle reproduction paradigms [63,64]. One study employed laboratory-based fatigue protocols, as described in Schmitz et al. [52], where participants performed cycling or leg press exercises to exhaustion, followed by weight-bearing simulations using axial loads to assess pre- and post-fatigue biomechanics.

#### 3.3.4. Device Calibration Procedures

Calibration procedures were inconsistently reported across studies, which poses a significant challenge for reproducibility and cross-study comparisons. Among the studies that reported calibration, approaches included static poses, such as the N-pose, orthostasis, or motionless standing, to define anatomical neutral positions [35,36,37,39,40,46]. Others employed standard static single-leg body-weight calibration [58,59,60,62]. Some studies also employed device-specific resets or zeroing procedures before each test [43,65]. Others implemented more comprehensive calibration routines—for example, Bellitti et al. [26] calibrated IMUs to identify offset, scale factor, and misalignment matrix relative to the device axes. In contrast, several studies—particularly those conducted in preoperative or intraoperative contexts—did not report any calibration procedures. This inconsistency in calibration reporting presents a significant barrier to reproducibility and cross-study comparison.

### 3.4. Outcome Measures and Validation

A wide range of wearable-derived outcomes were captured across the studies, spanning four categories: laxity tests, joint kinematics, loading proxies/kinetics, and temporospatial proxies. Validation approaches varied from in-study comparisons against criterion references (e.g., optical motion capture, force plates, bone pins, arthrometers) to reliance on previously published validity or not reporting any validation. A summary of outcome measures for each study is available in Table 3, with details in the Supplementary Material File S1.

#### 3.4.1. Laxity Tests

Tibiofemoral laxity was quantified during manual tests using IMUs, electromagnetic sensors, image analysis of tibial motion, or brace-embedded sensors. Electromagnetic systems have the strongest criterion evidence from a cadaveric bone–pin comparison, which demonstrated near-perfect correlation (Pearson’s correlation r = 0.995) and sub-millimetre translation error (=0.85 mm) [57]. The same system was later applied clinically by Yagi et al. [55] and Kuroda et al. [54]. Subsequent intra-operative and clinical studies focused on posterior tibial linear acceleration and related measures, such as coupled anterior tibial translation (c-ATT) and the acceleration of posterior translation (APT), to characterise the reduction phase of the pivot-shift [47,54]. Labbé et al. further indicated that tibial translation velocity and acceleration correlate most with clinical grades [53].

Brace-based systems that integrate IMUs with stretch sensors compared knee angles against optoelectronic motion capture (errors varying by plane), whereas anterior–posterior translation in the brace was only cross-checked against clinical Lachman grading rather than motion capture [26]. A dedicated anterior tibial translation device, consisting of two inductive displacement sensors, showed good agreement with a Lachmeter reference in both loaded and unloaded conditions [65]. Image-analysis approaches and smartphone-IMU applications are also used clinically to characterise pivot-shift behaviour, typically relying on clinician grades or previously published validity rather than new criterion comparisons in the same cohort [18,42,49]. Some studies additionally report reliability or associations with clinical grading. This information is informative but conceptually distinct from criterion accuracy.

#### 3.4.2. Joint Kinematics

Knee flexion, abduction/valgus, and internal rotation were usually estimated with wearable motion sensors across landings, cutting, gait, or stair tasks. The strongest criterion evidence shows excellent agreement for knee flexion and good agreement for frontal and transverse angles versus optical motion capture: for example, knee flexion reached intraclass correlation coefficients (ICCs) = 0.99 with continuous RMSE of around 1.1°, and peak flexion showed Bland–Altman limits from around −1.2° to +2.7°; abduction and internal rotation errors were larger but still small (RMSE between 2.6° and 2.9°; peak abduction limits from around −8° to 6.9°) [39].

Beyond discrete angles, some studies present waveform-based kinematics. For example, Buttton et al. reported knee joint angle waveforms across tasks rather than only discrete metrics [37]. Nonlinear analyses have also been explored: Albano et al. derived the maximal Lyapunov exponent from the knee flexion–extension signal during gait to characterise movement variability after ACL reconstruction [40].

Finally, joint-position sense (JPS) has been assessed using inertial sensors and electrogoniometers as an angle-reproduction error during active tests [43,44,63,64]. Validation in this area is largely methodological, emphasising repeatability and prior literature, rather than new comparisons to a gold standard.

#### 3.4.3. Loading Proxies from Insoles and Model-Estimated

Force-sensing insoles (loadsol) provided peak impact force (PIF), loading rate (instantaneous/average), impulse, and limb symmetry indices (LSIs) during hop, landing, cutting and gait. Criterion comparisons against force plates demonstrated good to excellent agreement at 200 Hz (ICCs ranging from 0.76 to 0.99) and more variable agreement at 100 Hz [62]. Several studies relied on this prior validity without new in-study testing [58,59,60,61]. A few on-field studies utilised IMU kinematics to predict knee abduction moment (KAM) and compared these predictions with laboratory-measured KAM. High/low KAM classification reached about 73–80% accuracy (area under the curve AUC = 0.81–0.85), and regression explained 33–46% of the variance [36]. These figures describe model-to-reference agreement rather than a direct accuracy test of the wearable sensor. In a separate feedback study, changes in a wearable feature (thigh coronal angular velocity) were correlated with changes in KAM (R^2^ = 0.55), indicating a relationship but not a criterion validation of the wearable [48].

#### 3.4.4. Validated Temporospatial Proxies

We included temporospatial metrics as proxy knee outcomes only when they were ACL-task-specific and validated against a reference standard. Ahmadian et al. quantified foot-ground initial/terminal contact instances, flying/landing times, and hop distance/LSI during triple single-leg hops with small errors against motion capture/tape (e.g., IC = 2 [22] ms; total progression = 2.4% [1.0–4.0%]) [46].

#### 3.4.5. Patient-Reported Outcome Measures (PROMs)

PROMs, such as International Knee Documentation Committee Subjective Knee Form (IKDC-SKF), Knee Injury and Osteoarthritis Outcome Score (KOOS), and ACL–Return to Sport Index (ACL-RSI), were used to contextualise wearable-derived measures across several studies. IMU-based functional tests showed moderate associations with PROMs early after ACLR. For example, IKDC-SKF versus vertical jump/side-hop/Y-balance typically fell in Spearman’s rank correlation (r_s) between 0.37 and 0.58 at 3–6 months [44]. Ahmadian et al. reported a moderate to strong correlation (r = 0.40–0.66) between temporospatial metrics in the hop-task and KOOS [46]. In contrast, gait asymmetry from insoles showed weak relationships with IKDC score at 6 months (r below 0.26) [58], whereas jump-landing insole asymmetry showed small to moderate relationships with IKDC and ACL-RSI (impulse LSI r = 0.34–0.37) [58]. Diermeier et al. found no significant correlations between kinematic outputs obtained during the pivot-shift manoeuvre [49].

### 3.5. Descriptive TRL Mapping

Using the descriptive TRL mapping in Table 1, most devices clustered at prototype, TRL 3–4, or research deployment/initial clinical studies, TRL 5–6. Only one device [43] reached workflow trials and multi-site pilots, TRL 7, and none reached regulatory approval and integration, TRL 8–9. Levels 1–2 were out of scope since eligibility required a functioning wearable system that produced quantitative knee outcomes. A summary of assigned TRL bands for each study, with justifications, is provided in Table 4.

## 4. Discussion

This scoping review provides a synthesis of how wearable devices have been used to assess knee joint outcomes following ACL injury or reconstruction. While these studies demonstrate innovative device designs and applications, their translational maturity varies widely. To evaluate clinical readiness, we applied a context-adapted technology readiness level (TRL) framework and conducted a narrative appraisal of methodological rigour and validation practices. This approach highlights not only what outcomes are being measured but also how rigorously, and where the field currently stands in terms of clinical integration. Beyond listing devices and protocols, we compare device types, draw task-specific recommendations on sampling frequency, and identify protocol elements (e.g., calibration and trial averaging) that most influence reliability. We also distinguish between directly measured outcomes and model-estimated surrogates, which is critical for clinical interpretation.

### 4.1. Synthesis by Device Type

IMUs were the most commonly used, given their portability and ability to capture multiplanar kinematics. Their strengths include versatility (usable in lab or field), wireless data collection, and good accuracy for joint angles in controlled tasks. For example, IMU-based knee angle estimates have shown excellent agreement with gold-standard motion capture (knee flexion angle ICC = 0.99 with RMSE = 1.1°), and slightly larger but acceptable errors (RMSE = 2.6–2.9°) for abduction and internal rotation [39]. This makes IMUs well-suited for gait, jump-landings, and cutting manoeuvres. However, IMUs are susceptible to soft-tissue artefacts and drift over time. They require careful calibration and alignment to anatomical axes, which was often omitted in the reviewed studies, potentially impacting accuracy.

Accelerometers require minimal setup and computation, and have been used as low-cost surrogates for the reduction phase of the pivot-shift manoeuvre [49,50,51]. Lopomo et al. strapped an accelerometer to the tibia during pivot shift, detecting distinct acceleration spikes that corresponded to knee instability [51]. However, without rotational data, they cannot characterise full 3D kinematics.

Electromagnetic sensors were applied intraoperatively and in clinical exams to characterise pivot-shift mechanics [54,55,57]. Kubo et al. demonstrated an electromagnetic system could measure tibial translation with sub-millimetre error (0.85 mm) and near-perfect correlation (r = 0.995) against a bone–pin gold standard [57]. However, their weaknesses include sensitivity to metal interference and the required tethered setups. This limits usability in dynamic or cluttered environments outside the lab or operating room. None of the electromagnetic-based approaches were integrated into routine practice, despite technical success in research settings.

Force-sensing insoles are a promising tool for kinetic assessment, particularly for tasks like gait, jump-landing, and hop tests. Across landing and hop tasks, the insoles show good-to-excellent agreement with force plates when data are sampled at 200 Hz and normalised with consistent body-weight calibration. In contrast, at 100 Hz, validity is more variable, with a slight underestimation bias for peak forces and loading rates [60,62]. In ACLR cohorts, insole outcomes (PIF, loading rates, impulse) provide repeatable LSIs but relate only modestly to PROMs, highlighting that mechanics and symptoms capture different constructs [58,59]. They offer bilateral coverage, low setup cost and real-world feasibility. However, they provide only the vertical force component and are sensitive to footwear and sampling rate [60,61].

Electrogoniometers offer simple, high-rate (4kHz) angle capture adequate for JPS tasks. They were often paired with electroencephalography (EEG) and surface electromyography (sEMG), which added neurophysiologic context to proprioception during early recovery [63,64]. However, they are limited by a single-plane measurement and soft-tissue artefact. An inductive displacement sensor system for Lachman testing measured anterior tibial translation and showed strong concurrent validity (ICC > 0.89; MDC 1.67–1.93 mm) against a standard Lachman arthrometer [65].

Therefore, device choice should match the clinical question. IMUs are recommended for multi-planar kinematics (sagittal angles are most accurate), while a single tibial accelerometer is used for clinical or intra-operative pivot-shift grading. Force-sensing insoles are proffered for bilateral loading and asymmetry, and electromagnetic trackers for the highest-accuracy laxity under controlled, wired setups.

### 4.2. Sampling Frequency

Sensor sampling frequency emerged as a critical technical parameter influencing the quality of the data. The included studies spanned a wide range of sampling rates, from as low as 40 Hz up to several kilohertz, with some not reporting their rates at all. Generally, higher-frequency sampling allows capture of rapid transient events (at the cost of more data and potential noise), whereas too low a rate risks missing peak values or timing events. Based on the collective evidence, the necessary minimum sampling rate depends on the task being measured and the signal of interest.

For high-impact, rapid events, such as the pivot shift jerk or the impact peak during a jump landing, a relatively high sampling rate is required to capture the amplitude and timing of spikes accurately. Evidence from the insole validation study by Peebles et al. supports this: at 100 Hz, the insoles underestimated true peak forces and showed more variable agreement with force plates, whereas at 200 Hz they achieved much tighter agreement (ICC of around 0.99 for peak force) [62]. Accordingly, studies that measured landing impact metrics often used a sampling frequency of 200 Hz when possible [60]. We can therefore conclude that 200 Hz is a suitable minimum for impact kinetics. Similarly, for the pivot shift acceleration, earlier studies using 40–60 Hz electromagnetic sensors did characterise the event. However, higher-frequency data (Labbé et al. sampled IMUs at 150 Hz) likely captures the acceleration profile more accurately, including the precise peak and any high-frequency oscillations as the tibia reduces [41]. Though no study directly compared sampling rates for pivot shift, it is reasonable to recommend 100 Hz or greater for quantifying the pivot shift acceleration or velocity, given the sub-second nature of the event.

For moderate-speed motions like gait, squats, or stair climbing, lower sampling can be sufficient. The gait cycle occurs over a relatively long (stance phases last around 0.6–0.8 s), so even 50 Hz can capture key gait events with acceptable resolution [81]. Indeed, some gait/stair studies used 60 Hz IMUs (e.g., Di Paolo et al. at 60 Hz for a multi-IMU gait/cutting analysis) and reported meaningful results on knee angles [35]. In those contexts, the signals (joint angles, smooth acceleration patterns) are lower-frequency, and 50–100 Hz is generally adequate. None of the included studies reported adverse effects of 50–60 Hz on gait or cut kinematics measurements. Therefore, 50 Hz appears acceptable as a minimum for moderate-speed movements. However, if temporospatial parameters, such as initial contact detection, are needed, higher rates improve resolution.

For joint position sense testing, the electrogoniometer studies were sampled at 4 kHz to synchronise with neurophysiological recordings and maximise resolution. In terms of measuring joint angle, such high rates are unnecessary, as even 20 Hz would capture a deliberate repositioning task. Thus, the 4 kHz usage was likely to match EEG or other signals.

### 4.3. Measurement Protocols and Outcome Metrics

Across the included studies, instrumented pivot-shift metrics, especially posterior tibial acceleration during the reduction phase, correlated with surgeon grading and differentiated low from high-grade instability [41,50,51,54,55]. For anterior–posterior laxity, instrumented Lachman tests quantify anterior tibial translation in millimetres with high repeatability (ICC > 0.89; MDC around 1.7–1.9 mm) and are straightforward to interpret clinically [65]. Kubo et al. showed that an electromagnetic tracking system agrees closely with bone pins (near-perfect correlation, sub-millimetre error) [57]. Therefore, electromagnetic systems can serve as a practical, non-invasive criterion reference for validating wearable measures. Hence, we recommend a compact laxity set consisting of Lachman translation measurement and pivot-shift posterior tibial acceleration as the primary dynamic metric.

For high-impact tasks (drop landings, DVJ, hops), force-sensing insoles provide peak impact force (PIF) and impulse that are most informative when expressed as limb-symmetry indices (LSI). At 200 Hz, these measures agree closely with force plates and are repeatable; consistently revealing persistent off-loading at 6 months and graft-related differences [59,60,62]. IMU-derived knee flexion at landing is highly accurate (RMSE around 1°) and clearly reflects landing strategy (greater flexion indicates a softer landing) [39]. Frontal- and transverse-plane angles remain informative for valgus/rotation patterns but are measured less precisely with IMUs, and they should be interpreted with more caution [39]. Model-based surrogates (e.g., field KAM classification; vGRF/KEM estimated from IMUs) help track within-athlete trends but are not yet reliable enough for clinical decision-making [36,45,48]. We recommend pairing insole PIF/impulse as LSI (sampled at ≥ 200 Hz) with IMU knee flexion in DVJ/hops.

By mid-rehab, level gait asymmetries are small and correlate only modestly with PROMs. Hence, they are most useful for routine monitoring rather than decision-making [58]. Stair navigation can still expose early residual kinematic deficits (reduced flexion and excursion) [34]. Active JPS error is feasible and clinically interpretable. Used alongside functional tests, it can identify proprioceptive deficits for targeted rehabilitation [63,64].

Taken together, protocols that quantify mechanical stability (Lachman translation and pivot-shift acceleration) and dynamic loading or movement quality (PIF/impulse as LSI, IMU knee flexion) are the most reliable, interpretable, and ready for broader use. Model-based surrogates and temporospatial parameters serve as complementary monitors rather than primary decision indicators.

### 4.4. Methodological Gaps

Across studies, three recurring methodological themes emerged: limited direct validation, underreporting of calibration and trial handling, and infrequent justification of sample size or sex-stratified analysis.

Only 11 of the 32 studies performed in-study validation of their devices, while 15 cited prior literature only, and 6 studies provided no validation data at all. This lack of transparency undermines confidence in the measurements and limits the ability to make meaningful comparisons between different wearable systems. Moreover, validation should extend beyond technical details. Integrating PROMs alongside traditional validation metrics would provide a patient-centred perspective on recovery outcomes. Therefore, future studies should implement the following:

- Systematic validation protocols against gold-standard systems.
- Transparent reporting of accuracy metrics and statistical agreement.
- Use outcome-specific metrics tailored to the data type (e.g., RMSE for continuous variables, ICC for reliability, F1 score for classification tasks).
- Clear differentiation between validation accuracy, test–retest reliability, and predictive model performance.
- Inclusion of PROMs.

Establishing a standardised set of validation and accuracy reporting guidelines would significantly improve reproducibility, comparability, and clinical trust in wearable sensor data.

Further, calibration procedures were rarely reported in sufficient detail, despite their crucial role in ensuring accurate sensor-based measurements. Only a minority of studies described the use of static poses (e.g., N-pose or quiet standing), dynamic calibration, or device-specific resets. The absence of calibration details introduces uncertainty into the derived metrics and poses a major barrier to reproducibility. This inconsistency highlights the urgent need for consensus-driven calibration guidelines in ACL research. Similarly, most studies did not specify how repeated trials were handled (e.g., whether results were averaged, peak values were selected, or outliers were excluded), which limits the interpretability and comparability of their findings. Establishing and reporting trial repetition protocols, including the number of repetitions, selection methods (e.g., mean vs. best trial), and exclusion criteria, should become standard practice.

Moreover, participant characteristics were inconsistently reported across the studies, varying in the level of detail provided on sex, age, height, weight, BMI, and activity level. This limits the generalisability of findings and results across different patient groups. Furthermore, while exclusion criteria were generally consistent across studies, they were not always reported transparently. Future research should also adopt transparent and standardised participant reporting that includes sex, age, weight, height, BMI, activity level, and relevant clinical history (e.g., time since injury or surgery) and exclusion criteria to improve reproducibility and facilitate meaningful comparisons across cohorts. Additionally, despite well-documented evidence that females are at greater risk of ACL injury, only 1 study used a female-only cohort. In contrast, three studies recruited male-only groups. This imbalance raises concerns about the applicability of wearable devices to female populations, particularly considering the biomechanical and neuromuscular differences. Future research should ensure female representation is sufficient to reduce gender bias in both device development and recovery protocols.

### 4.5. Technology Readiness and Implementation Roadmap

Using descriptive TRL bands, half of the studies (*n* = 16) clustered within TRL bands 3–4, 15 at TRL 5–6, only one study achieved TRL 7, and none achieved TRL 8–9 (full integration and regulatory approval). Studies from TRL 3–4 indicated prototype maturity with evaluation primarily in research-led protocols. Advancing beyond this stage will require demonstration of routine clinical use and external validation in broader populations. Studies at bands 5–6 typically demonstrated task-specific validity and relevance to post-operative care (e.g., force-sensing insoles for loading symmetry in gait/landings; IMU-based measures for pivot-shift or functional kinematics) but were not yet embedded into routine pathways. This overall TRL analysis underscores that even the most advanced wearable systems remain in pre-integration stages, highlighting a significant gap between research prototypes and widespread clinical adoption.

### 4.6. Recommendations for Future Research

To support the clinical translation of wearable sensors in ACL injury assessment, the field should move towards greater standardisation, transparency, and methodological rigour. While existing frameworks such as STROBE (for observational studies) [82], TRIPOD (for prediction model development) [83], and SPIRIT-AI (for AI-integrated clinical trials) [84] offer valuable guidelines for study design and reporting, they do not fully address the unique methodological, technical, and clinical integration challenges posed by wearable sensor systems. These include device calibration, sensor drift, usability in dynamic environments, and integration into clinical workflows. As such, there is a clear need to develop dedicated reporting guidelines tailored to orthopaedic and rehabilitation-focused wearable devices.

Future studies should implement systematic validation protocols and clearly report accuracy metrics, using appropriate statistical methods to distinguish between accuracy, reliability, and predictive model performance. PROMs should be more consistently integrated alongside sensor-derived data to enable a comprehensive understanding of recovery. Standardising the selection, timing, and interpretation of PROMs alongside sensor-derived data enables objective metrics to be contextualised within the patient’s experience and functional recovery. Future studies should specify and report the method used to determine the sample size, using an approach appropriate to the study’s purpose. Furthermore, usability, compliance, and cost-effectiveness should be systematically evaluated across both clinic-based and home-based rehabilitation settings to ensure real-world viability. Longitudinal designs will be critical for linking sensor-derived measurements to meaningful clinical outcomes, such as reinjury risk, RTS readiness, and long-term joint health. By addressing these methodological and translational gaps, future research can help realise the full potential of wearable devices to enhance ACL rehabilitation, personalise care pathways, and support evidence-based decision-making.

### 4.7. Limitations

While this review benefited from clinical expertise within the author team, including an orthopaedic surgeon, it did not involve a formal stakeholder consultation process with broader groups, such as patients, physiotherapists, or technology developers. As such, some practical or user-centred perspectives may not have been fully captured. Furthermore, exclusion of non-English studies may have introduced language bias. Finally, our TRL mapping used adapted bands and qualitative judgements at the device/system level. These assignments depend on what each study reported and should be interpreted as indicative maturity rather than definitive rankings. Nonetheless, this work provides a valuable roadmap for advancing the development, validation, and clinical adoption of wearable devices in the management of ACL injuries.

## 5. Conclusions

The findings highlight the growing diversity and adaptability of wearable devices in capturing clinically relevant biomechanics from intraoperative assessments to home-based rehabilitation. IMUs are the most commonly used wearable devices in ACL research and are currently the most practical option for multi-planar kinematics in both laboratory and field settings, with sagittal angles typically being the most accurate. However, careful calibration is needed to limit drift and soft-tissue artefact. Standalone accelerometers are well-suited to intraoperative or clinic-based pivot-shift grading, where a single tibial sensor can capture the reduction-phase spike, but they cannot recover full 3D motion. Force-sensing insoles are preferred for bilateral loading and asymmetry (e.g., landing and gait LSI). Their accuracy improves at 200 Hz; however, outputs are limited to the vertical component and are influenced by footwear. Electromagnetic trackers provide the highest spatial accuracy for laxity characterisation under controlled conditions, but sensitivity to metal and the need for wired hardware limit deployment beyond research settings.

However, despite the potential of wearable devices, their clinical translation remains limited. Key barriers include inconsistent validation against gold-standard systems, poorly reported calibration and testing protocols, and a lack of standardised outcome measures. The introduction of a TRL mapping in this review offers a novel approach to evaluate the maturity and translational potential of wearable applications in ACL care. Most applications sit at intermediate TRLs (3–6), reflecting pre-integration maturity and the need for larger clinical studies, workflow integration, and regulatory pathways before clinical adoption.

The field should now prioritise task-specific, interpretable metrics that map to clinical questions such as readiness to progress rehabilitation or RTS. Studies should justify sampling by task, standardise calibration, validate against a reference standard, and pair sensor outputs with PROMs. Multi-site trials and usability testing in the clinic and at home will accelerate translation. If these steps are followed, wearables can move from promising prototypes to tools that support clearer decisions and better long-term knee health.

## Figures and Tables

**Table 1:** Adapted TRL framework for wearable devices in ACL injury or reconstruction research.

TRL Band *	Category	Criteria
3–4	Prototype	Researcher-run; laboratory setting; healthy volunteers or small early patient pilots; no clinical workflow; custom pipeline; no regulatory status.
5–6	Research deployment/ initial clinical studies	Patients in clinical settings; clinician-performed/supervised; standardised protocol; research deployment; outputs not used to guide care; no regulatory claim.
7	Workflow trials/ multisite pilots	Operational use embedded in clinical workflows; multi-site/registry with standard operating procedure; routine rehab/clinic sessions; trained staff; evidence that outputs are used within the care pathway.
8–9	Regulatory approval and integration	Regulatory clearance and limited/routine integration into standard care; post-market/real-world evidence.

* Levels 1–2 were out of scope (no functioning wearable system).

**Table 2:** A summary of device details for each study (sorted by device type and year) [34,35,36,37,38,39,40,41,42,43,44,45,46,47,48,49,50,51,52,53,54,55,56,57,58,59,60,61,62,63,64,65]; NR = not reported. Hz = Hertz; kHz = kilohertz.

Year	Study	Device Type	Placement	Number of Devices	Sampling Rate	Data Processing Software
2025	Yona et al. [34]	IMU (9 axis)	- Pelvis, - Thighs, - Shanks, - Feet	7	NR	IBM SPSS (Version 29)
2024	Di Paolo et al. [35]	IMU (9 axis)	- Upper limbs, - trunk, - Lower limbs	15	60 Hz	Dedicated Xsens software and a custom MATLAB script (The MathWorks, Natick, MA, USA)
2024	Benjaminse et al. [36]	IMU (9 axis)	- Head (right side), - Sternum, - Hands (posterior side), - Wrists (dorsal side), - Above elbows (lateral side), - Scapula spines (middle), - Posterior superior iliac spines (middle), - Thighs (lateral side), - Tibias (medial surface), - Forefoot (dorsal side)	17	240 Hz	Custom MATLAB script vR2022a (The MathWorks, Natick, MA, USA)
2022	Button et al. [37]	IMU (9 axis)	- Upper thighs (centrally and halfway between the greater trochanter and lateral epicondyle), - Lower legs (proximal medial surface of each tibia), - Feet (dorsum of each foot), - Sacrum	7	NR	MVN BIOMECH studio software (version 4.4), custom written MATLAB code (MATLAB version 2015a; The MathWorks Inc., Natick, MA, USA)
2022	Bellitti et al. [26]	IMU (9 axis) + stretchable strain sensors	IMUs: - Proximal anterolateral part of the tibia, - Distal lateral part of the femur Stretch sensors: - Lateral side of the knee, - Femur epicondyle, - Anterior aspect of the knee	IMUs: 2 stretch sensors: 3,	40 Hz	Custom software (written in LabVIEW2017, National Instruments, Austin, TX, USA), MATLAB (MathWorks)
2022	Baldazzi et al. [38]	IMU (9 axis)	- Medial upper portion of the tibial crest, - Foot dorsum	2	500 Hz	Custom MATLAB scripts and functions
2021	Fan et al. [39]	IMU (9 axis)	2 per leg: - Thigh, - Shank	4	100 Hz	Custom
2021	Albano et al. [40]	IMU (9 axis)	- Thighs (one on each leg), - Shank (one on each leg)	4	60 Hz	Custom MATLAB script, UNO Biomechanics Nonlinear Analysis Toolbox
2015	Labbé et al. [41]	IMU (9 axis)	- Tibia, - Femur	2	150 Hz	Custom
2024	Portillo-Ortiz et al. [42]	Smartphone (accelerometer + gyroscope)	- Tibial tuberosity (2 fingers below patella, incline towards medial aspect of tibia)	1	NR	Neural-network classification via the app; features processed in MATLAB; CSV assembled with a Python script
2023	Niederer et al. [43]	IMU (6 axis)	- Tibia (highest circumference of the lower leg)	1	4.5 kHz–9.0 kHz	NR
2023	Mengis et al. [44]	IMU (6 axis)	- Tibial tuberosity	1	NR	Orthelligent HOME app
2023	Sun et al. [45]	IMU (6 axis)	- Chest (trunk-fifth thoracic vertebrae), - Waist (pelvis-mid-point between left and right anterior superior iliac spine), - Right and left thigh (thigh-midpoint between the left anterior superior iliac spine and left femur medial epicondyle), - Right and left shank (shank-one-third point between left femur medial epicondyle and left tibia apex of medial malleolus near proximal end of tibia), - Right and left foot (second metatarsal)	8	100 Hz	Keras (v2.5.0), TensorFlow (v2.5.0)
2020	Ahmadian et al. [46]	IMU (6 axis)	Physilog BFSr-3: - Forefoot, - Upper shank (criterion-related validity), Physilog 5: Bilaterally: - Feet, - Shanks (construct validity)	Physilog BFSr-3: 2 Physilog 5: 4	500 Hz (criterion-related validity), 256 Hz (construct validity)	Custom
2020	Kawanishi et al. [47]	IMU (6 axis)	- Tibia (between the lateral aspect of the anterior tibial tuberosity and Gerdy tubercle)	1	NR	NR
2012	Dowling et al. [48]	IMU (6 axis)	- Chest, - Thigh, - Shank	3	240 Hz	Custom real-time feedback
2020	Diermeier et al. [49]	Accelerometer + image-analysis system (for lateral compartment translation)	- Tibia (Gerdy tubercle)	1	120 Hz	Specifically developed application
2018	Musahl et al. [50]	Accelerometer	- Proximal tibia	1	NR	Proprietary iPad software (KiRA acceleration); custom iPad Image Analysis (video-based translation)
2012	Lopomo et al. [51]	Accelerometer	- Tibia	1	NR	Klee—dedicated software used for kinematic analysis for the BLU-IGS navigation system
2015	Schmitz et al. [52]	Electromagnetic sensor	- Lateral thigh (midpoint), - Centre of patella, - Tibial shaft (midpoint)	3	100 Hz	NR
2010	Labbe et al. [53]	Electromagnetic sensor	- Thigh - Shank	2	NR	Custom software developed in MATLAB (Mathworks, Natick, MA)
2008	Kuroda et al. [54]	Electromagnetic sensor	- Thigh (10 cm above the patella), - Tibia (7 cm below the tibial tubercle) (- attached to a stylus for digitising anatomical landmarks)	3 receivers and 1 transmitter	60 Hz	6 Degrees of Freedom (DOF) tibiofemoral kinematics derived from the joint coordinate system [56]
2007	Yagi et al. [55]	Electromagnetic sensor	- Tibia (10 cm below tibial tubercle), - Femur (13 cm above patella)	2	60 Hz	6 Degrees of Freedom (DOF) tibiofemoral kinematics derived from the joint coordinate system [56]
2007	Kubo et al. [57]	Electromagnetic sensor	- Thigh (1), - Tibia (1—distal to tibial tubercle, 1- proximal to the ankle)	3 (+ 1 additional sensor to register anatomical reference points)	40 Hz	NR
2025	Cherelstein et al. [58]	Force-sensing insole	- Under foot	2 (1 for each foot)	100 Hz	Loadsol mobile application and custom processing program [62]
2025	Cherelstein et al. [59]	Force-sensing insole	- Under foot	2 (1 for each foot)	100 Hz	Loadsol mobile application and custom processing program [62]
2021	Luftglass et al. [60]	Force-sensing insole	- Under foot	2 (1 for each foot)	200 Hz (for 33 participants), 100 Hz (for 7 participants)	Load analysis program (LAP): a custom MATLAB user-interface for loadsol^®^ data.
2019	Peebles et al. [61]	Force-sensing insole	- Under foot	2 (1 for each foot)	100 Hz	NR
2018	Peebles et al. [62]	Force-sensing insole	- Under foot	2 (1 for each foot)	2 types: 100 Hz, 200 Hz	MATLAB (Version 9, The Mathworks, Inc, Natick, MA, USA)
2025	Nyffenegger et al. [63]	Electrogoniometer	- Knee (centre aligned with knee joint; proximal arm to greater trochanter, distal arm to lateral malleolus)	1	4000 Hz	IMAGO Process Master (Pfitec^®^, Endingen, Germany), Microsoft^®^ Excel spreadsheet (Windows 10, Microsoft Corporation, Redmond, WA, USA).
2024	Busch et al. [64]	Electrogoniometer	- Knee (centre aligned with knee joint; in the midline between the lateral femoral and tibial epicondyle of the leg)	1	4000 Hz	IMAGO Process Master (Pfitec^®^, Endingen, Germany), Microsoft^®^ Excel spreadsheet (Windows 10, Microsoft Corporation, Redmond, WA, USA).
2024	Deiss et al. [65]	Inductive displacement sensor	- Patella, - Tibial tuberosity	2	NR	NR

**Table 3:** Wearable-derived outcome measures by study, grouped by measurement category and validation reference. Validation reference codes: I—in-study criterion comparison; P—prior literature cited for validity with no in-study criterion; N—not reported.

Category	Study	Validation Reference	Device Type	Outcome Measure
Laxity tests	Lopomo et al. [51]	I	Accelerometer	Three-dimensional acceleration of tibia
	Kubo et al. [57]	I	Electromagnetic sensor	Tibial posterior translation, tibial lateral translation, max posterolaterally directed velocity
	Yagi et al. [55]	I	Electromagnetic sensor	Tibial linear acceleration
	Labbé et al. [41]	I	IMU (9 axis)	Tibial velocity spike, femoral velocity spike, tibial acceleration drop, femoral acceleration drop
	Bellitti et al. [26]	I	IMU (9 axis) + stretchable strain sensors	Anterior–posterior translation, medial–lateral translation, internal–external rotation, flexion–extension rotation, adduction–abduction rotation
	Deiss et al. [65]	I	Inductive displacement sensor	Anterior tibial translation
	Musahl et al. [50]	P	Accelerometer	Lateral-compartment tibial translation, lateral-compartment tibial acceleration
	Diermeier et al. [49]	P	Accelerometer + image-analysis system	Anterior tibial translation (video analysis), tibial acceleration
	Kuroda et al. [54]	P	Electromagnetic sensor	Coupled anterior tibial translation (c-ATT), acceleration of posterior translation (APT)
	Portillo-Ortiz et al. [42]	P	Smartphone (accelerometer + gyroscope)	Angular velocity (rotational laxity measurement) 3 axis
	Labbe et al. 2010 [53]	N	Electromagnetic sensor	AP/ML/total tibial translation magnitude, tibial internal–external rotation magnitude, tibial adduction–abduction magnitude, AP/ML/total translation velocity, tibial internal–external rotation angular velocity, tibial adduction–abduction angular velocity, AP/ML/total translation accelerations, tibial internal–external rotation angular acceleration, tibial adduction–abduction angular acceleration
	Kawanishi et al. [47]	N	IMU (6 axis)	Tibial external rotational angular velocity, tibial acceleration
Joint kinematics	Fan et al. [39]	I	IMU (9 axis)	Knee internal rotation, knee abduction, knee flexion
	Busch et al. [64]	P	Electrogoniometer	Knee joint angle (absolute error, constant error, variable error)
	Nyffenegger et al. [63]	P	Electrogoniometer	Knee joint angle (absolute angular error) (JPS)
	Mengis et al. [44]	P	IMU (6 axis)	Knee displacement (mm), extension/flexion angles, knee displacement (degrees), angle reproduction angle (JPS)
	Niederer et al. [43]	P	IMU (6 axis)	Knee displacement, angle reproduction error (JPS)
	Yona et al. [34]	P	IMU (9 axis)	Knee flexion angle
	Di Paolo et al. [35]	P	IMU (9 axis)	Knee flexion angle, knee valgus angle
	Baldazzi et al. [38].	P	IMU (9 axis)	Tibial angular displacement—root mean square (RMS) of angular velocity (foot and leg), peak angular velocity (foot and leg), RMS of acceleration (foot and leg), sway path (tibia), sway area (tibia), sway area eccentricity (tibia)
	Schmitz et al. [52]	N	Electromagnetic sensor	Anterior tibial translation, knee-flexion excursion, peak knee-flexion angular acceleration (secondary outcome)
	Dowling et al. [48]	N	IMU (6 axis)	Max knee flexion angle, first peak of thigh coronal angular velocity
	Albano et al. [40]	N	IMU (9 axis)	Maximal Lyapunov exponent (LyE) of knee flexion–extension angle
	Button et al. [37]	N	IMU (9 axis)	Knee joint angle waveforms in sagittal and frontal planes
Loading proxies/kinetics	Peebles et al. [62]	I	Force-sensing insole	Peak impact force (PIF), loading rate (LR), impulse (IMP) (total force applied over time), limb symmetry index (LSI) for IP/LR/IMP
	Sun et al. [45]	I	IMU (6 axis)	Vertical ground reaction force (vGRF), external knee extension moment (KEM)
	Benjaminse et al. [36]	I	IMU (9 axis)	Knee abduction moment (KAM) class (classification models), peak KAM (regression models)
	Peebles et al. [61]	P	Force-sensing insole	Peak impact force (PIF), loading rate (LR), impulse (IMP) (total force applied over time), LSI for IP/LR/IMP
	Luftglass et al. [60]	P	Force-sensing insole	Peak impact force, impulse (total force applied over time), LSI derived from the above metrics
	Cherelstein et al. [58]	P	Force-sensing insole	Peak impact force (PIF), instantaneous loading rate (ILR), average loading rate (ALR), impulse (area under the force–time curve from heel strike to toe-off, LSI derived from the above metrics
	Cherelstein et al. [59]	P	Force-sensing insole	Peak impact force (PIF), average loading rate, impulse, LSI derived from the above metrics
Validated temporospatial proxies	Ahmadian et al. [46]	I	IMU (6 axis)	Foot-ground initial contact (IC) instants, foot-ground terminal contact (TC) instances, flying and landing times, individual distances, foot forwards progression distances, time and distance-based LSI

**Table 4:** A summary of TRL bands for each study with justification.

Study	TRL Band	Justification
Niederer et al. [43]	7	Nationwide rehabilitation registry using Orthelligent Pro tibial IMU with standard operating procedure-based, clinician-run testing (hop/jump, Y-Balance, JPS) across multiple centres; operational use in practice, but no explicit regulatory status.
Yagi et al. [55]	5–6	Pivot-shift under general anaesthesia at 1-year follow-up using thigh/shank Polhemus Fastrak sensors; validated pipeline (r = 0.995; ≤0.85 mm error); research deployment in a clinical setting, not routine care.
Kuroda et al. [54]	5–6	Intraoperative pivot-shift under GA using strapped FASTRAK sensors and stylus digitisation; 6-DoF kinematics with c-ATT/APT outcomes; patient cohorts in theatre; research workflow, no routine integration.
Lopomo et al. [51]	5–6	Intra-operative pivot-shift under general anaesthesia using strapped KiRA accelerometer validated against navigation; good repeatability/correlation; single-site research setup, not routine care.
Musahl et al. [50]	5–6	Multi-centre, surgeon-performed preoperative pivot-shift under general anaesthesia using tibial IMU (KiRA) and iPad image-analysis; trained users; research setting; no routine workflow/regulatory use.
Kawanishi et al. [47]	5–6	Intraoperative pivot-shift under general anaesthesia with a strapped tibial IMU quantifying acceleration and external rotation angular velocity; 91 ACLR cases, surgeon-run protocol, receiver operating characteristic cut-offs for predicting residual instability; research workflow, no routine integration/regulatory status.
Diermeier et al. [49]	5–6	Multi-centre (4 sites) pivot-shift quantified preoperatively, at time zero after ACLR (under general anaesthesia) and after 24 months using a strapped KiRA accelerometer and a tablet image-analysis; surgeon-standardised protocol; research deployment (no routine workflow/regulatory status).
Labbe et al. [53]	5–6	Clinician-performed pivot shift in ACL-deficient patients using thigh/shank electromagnetic sensors; multi-surgeon cohort; custom MATLAB feature extraction/PCA; research workflow, no routine clinical integration.
Labbé et al. [41]	5–6	Clinical pivot-shift in ACL-deficient patients (no GA) using two strapped IMUs (tibia and femur); “femoral acceleration drop” strongly matches clinical grade (r = 0.84); research use, not routine.
Kubo et al. [57]	5–6	Clinic pivot-shift in ACL-deficient patients using Polhemus Fastrak; correlated with IKDC; bone–pin validation (r = 0.995, ≤0.85 mm error); researcher-run, single-site
Portillo-Ortiz et al. [42]	5–6	Multi-centre outpatient clinics; trained surgeons; immediate feedback; framed as trial version; not yet routine/regulated integration.
Button et al. [37]	5–6	Used in ACLR patients within a physiotherapy department; seven IMUs with a custom MATLAB reporting tool (gait/squat/stair ascent); clinician-run agreement study; complete research workflow but not embedded as routine care and no regulatory status stated
Yona et al. [34]	5–6	ACLR patients tested on full staircase during clinic visits; complete workflow but not embedded as routine care; no regulatory claims.
Mengis et al. [44]	5–6	Single-centre clinical validation of a commercial tibial IMU during supervised rehabilitation visits at 3 and 6 months post-ACLR; standardised test battery (ROM, Y-Balance, vertical/side hops) with significant correlations to IKDC; conducted within an RCT; regulatory status not stated.
Cherelstein et al. [59]	5–6	Multi-site clinic-based DVJ testing at 6 ± 1 months post-ACLR using force-sensing insoles (100 Hz) with a standardised calibration; outcomes PIF, ALR, impulse (LSI); supervised clinical assessments within a study, no routine workflow/regulatory claims.
Cherelstein et al. [58]	5–6	Multi-site rehab-clinic/clinical-lab treadmill gait at 6 ± 1 months post-ACLR using loadsol insoles (100 Hz) with standardised calibration; outcomes = PIF, ILR, ALR, impulse (LSI); researcher-supervised, no stated regulatory status or routine workflow integration.
Peebles et al. [62]	3–4	Laboratory validity/repeatability study in healthy athletes using loadsol insoles during single-leg hop and stop-jump; compared to force plates (100 Hz and newer 200 Hz version: validity improves, but absolute loads are underestimated); researcher-run, no clinical workflow.
Luftglass et al. [60]	3–4	Laboratory study in healthy adults using loadsol (200 Hz) and the LAP MATLAB interface for landing kinetics/LSI; researcher-run, no clinic deployment or regulatory status.
Peebles et al. [61]	3–4	Research-run RTS testing with loadsol insoles (100 Hz) during single/triple/crossover hops in ACLR vs healthy; outcomes = LSI of impact peak, loading rate, impulse; no clinical workflow/regulatory use.
Fan et al. [39]	3–4	Laboratory validation in healthy adults using four Xsens IMUs (100 Hz) on thigh/shank during drop landing and 45° cutting; new two-step complementary filter + single-pose calibration; errors vs Vicon = 1.07° flexion, 2.87° abduction, 2.64° internal rotation; researcher-run, no clinic workflow or regulatory use.
Di Paolo et al. [35]	3–4	Laboratory screening in healthy athletes with 15-IMU Xsens and custom processing; change of direction/deceleration tasks; Vicon used only for moments; research workflow, no clinical integration/regulatory status.
Benjaminse et al. [36]	3–4	Lab model-development study in healthy youth female footballers using 17-IMU, motion capture and force plates during unanticipated sidestep cutting; ML classifies high vs low KAM (AUC 0.81–0.85); researcher-run workflow, no field/clinical deployment or regulatory status.
Sun et al. [45]	3–4	Laboratory model-development in healthy males using 8 IMUs with motion capture and force plates; modular LSTM estimates vertical ground reaction forces / knee-extension moment in real time for single/double-leg drop landings; researcher-run; no clinical workflow/regulatory status.
Ahmadian et al. [46]	3–4	Laboratory setting, researcher-run pipeline; not embedded in routine care; no regulatory claims. Two foot/shank IMUs; validated IC/TC, times, and distances vs motion capture; exploratory patient–control cohort; KOOS correlations; no clinical workflow use.
Dowling et al. [48]	3–4	Laboratory drop-jump training in healthy athletes with 3 cabled IMUs and custom real-time feedback; Vicon/force plate used only as reference; researcher-run, no clinical workflow/regulatory status.
Baldazzi et al. [38]	3–4	Lab protocol in healthy soccer players using two strapped IMUs on tibia and foot during single-leg squat and crossover hop; reports reliability (ICC 0.29–0.84, MDC) for stability metrics; researcher-run, no clinical workflow/regulatory status.
Albano et al. [40]	3–4	Laboratory treadmill walking with four Xsens Dot sensors on thighs and legs; knee flexion–extension derived from inclinometry; Lyapunov exponent (LyE) variability metric; *n* = 4 (ACLR and healthy); researcher-run, no clinical workflow/regulatory status.
Busch et al. [64]	3–4	Laboratory JPS pilot with electrogoniometer, sEMG and dry-EEG (DSI-24); ACLR patients measured at 1.5/3–4/6 months; researcher-run, single-site; no clinical workflow or regulatory status.
Nyffenegger et al. [63]	3–4	Laboratory pilot, exploratory neurophysiology; small early postoperative cohort; no clinic workflow or deployment.
Schmitz et al. [52]	3–4	Lab VKLD test in healthy adults with strapped miniBIRD trackers measuring anterior tibial translation (ATT) and knee-flexion under 40% BW; researcher-run; no clinical workflow/regulatory status.
Bellitti et al. [26]	3–4	Smart knee brace (2 IMUs and 3 stretch sensors); lab characterisation vs Xsens plus small pilot (*n* = 4) of Lachman/drawer/pivot-shift vs optical; researcher-run; no clinical integration/regulatory.
Deiss et al. [65]	3–4	Laboratory prototype ATT device (two inductive displacement sensors at patella and tibial tuberosity) in healthy volunteers; good test–retest (Lachman ICC 0.90–0.94) and concurrent validity vs Lachmeter; researcher-run, no clinical integration/regulatory.

## Data Availability

The data supporting the findings of this study are available in Supplementary File S1 (Supplementary Materials).

## Supplementary Materials

The following supporting information can be downloaded at https://www.mdpi.com/article/10.3390/s25185837/s1, Supplementary File S1: Tables with detailed information on (by study): device, participants, test protocols, outcome measures and validation (.xlsx), Supplementary File S2: PubMed search strategy (.docx), Supplementary File S3: PRISMA-ScR checklist (.docx).

## Abbreviations

The following abbreviations are used in this manuscript:

ACF	Axial Compressive Force
ACI	Anterior Cruciate Insufficiency
ACL	Anterior Cruciate Ligament
ACL-RSI	ACL-Return to Sport Index
ACLD	ACL-Deficient
ACLI	ACL Intact
ACLR	Anterior Cruciate Ligament Reconstruction/Anterior Cruciate Ligament Reconstructed
ADL	Activities of Daily Living
ADLS	Activities of Daily Living Score
ALR	Average Loading Rate
AP/ML	Anteroposterior/Mediolateral
APT	Acceleration of Posterior Translation
ATP	Anterior Tibial Position
ATT	Anterior Tibial Translation
AUC	Area Under the Curve
BMI	Body Mass Index
BPTB	Bone–Patellar Tendon–Bone
BTB	Bone–Tendon–Bone
CAD	Computer-Aided Design
CHT	Crossover Hop Test
CKRS	Cincinnati Knee Rating System
DMD	Digital Medical Device
DOF	Degrees of Freedom
DVJ	Drop Vertical Jump
EMG	Electromyography
EPSRC	Engineering and Physical Sciences Research Council
GPT	Generative Pre-trained Transformer
GRF	Ground Reaction Force
HES	Hospital Episode Statistics
IC	Initial Contact
ICC	Intraclass Correlation Coefficient
ICRS	International Cartilage Repair Society
IDEAL	Idea, Development, Exploration, Assessment, Long-term Follow-up
IKDC	International Knee Documentation Committee
IKDC-SKF	International Knee Documentation Committee Subjective Knee Form
ILR	Instantaneous Loading Rate
IMP	Impulse
IMU	Inertial Measurement Unit
IPF	Impact Peak Force
IQR	Interquartile range
JPS	Joint Position Sense
KAM	Knee Abduction Moment
KEM	Knee Extension Moment
KFEXC	Knee-Flexion Excursion
KOOS	Knee Injury and Osteoarthritis Outcome Score
LAP	Load Analysis Program
LR	Loading rate
LSI	Limb Symmetry Index
LSTM	Long Short-Term Memory
MCID	Minimal Clinically Important Difference
MDC	Minimal Detectable Change
MEMS	Micro-Electromechanical Systems
MIMU	Magnetic Inertial Measurement Unit
MMAT	Mixed Methods Appraisal Tool
MRC	Medical Research Council
MRI	Magnetic Resonance Imaging
MVIC	Maximal Voluntary Isometric Contraction
N-pose	Neutral Pose
N/A	Not Applicable
NIRS	Near-Infrared Spectroscopy
NMES	Neuromuscular Electrical Stimulation
NR	Not Reported
OSF	Open Science Framework
PCA	Principal Component Analysis
PCC	Population, Concept, Context
PCL	Posterior Cruciate Ligament
PIF	Peak Impact Force
PRISMA	Preferred Reporting Items for Systematic Reviews and Meta-Analyses
PRISMA-2020	PRISMA 2020 Flow Diagram
PSM	Pivot-Shift Meter
QALY	Quality-Adjusted Life Year
QPS	Quantitative Pivot Shift
RCT	Randomised Controlled Trial
RMS	Root Mean Square
RMSE	Root Mean Square Error
ROM	Range of Motion
rRMSE	Relative Root Mean Square Error
RSI	Return to Sport Index
RTS	Return to Sport
SEM	Standard Error of Measurement
sEMG	Surface Electromyography
SLS	Single Leg Squat
SPIRIT-AI	Standard Protocol Items: Recommendations for Interventional Trials—Artificial Intelligence
STROBE	Strengthening the Reporting of Observational Studies in Epidemiology
SVM	Support Vector Machine
TAS	Tegner Activity Scale
TO	Toe-Off
TPR	True Positive Rate
TRL	Technology Readiness Level
TSLH	Triple Single-Leg Hop
UKRI	UK Research and Innovation
vGRF	Vertical Ground Reaction Force
VKLD	Vermont Knee Laxity Device
