# Supplementary material for: Wearable Devices for the Quantitative Assessment of Knee Joint Function After Anterior Cruciate Ligament Injury or Reconstruction: A Scoping Review
Source: Sensors (Basel). 2025 Sep 18;25(18):5837. doi: 10.3390/s25185837 (PMC12473230; doi:10.3390/s25185837)
Supplement: Supplementary file 1 [file sensors-25-05837-s001.zip › sensors-3824678-Supplementary File S2. PubMed search strategy.pdf]

Supplementary File S3. PubMed search strategy
Database/Platform: PubMed (MEDLINE via NLM)
Date executed: 27 August 2025
Search string (verbatim):

(ACL or "anterior cruciate ligament") and (injur* or reconstruct*) and (assess* or model* or measure* or estimat* or quantif*) and (wearable or sensor or sensors) and outcome

Limits/filters: None
